# Effectiveness of supervised Kegel exercises using bio-feedback versus unsupervised Kegel exercises on stress urinary incontinence: a quasi-experimental study

**DOI:** 10.1007/s00192-022-05281-8

**Published:** 2022-07-08

**Authors:** Donelle Cross, Nasreena Waheed, Michelle Krake, Daniel Gahreman

**Affiliations:** 1 College of Health and Human Sciences, Charles Darwin University, Darwin, Northern Territory 0909 Australia; 2 College of Nursing and Health Sciences, Flinders University, Bedford Park, 5042 Australia; 3 College of Nursing and Midwifery, Charles Darwin University, Darwin, Northern Territory 0909 Australia; 4 Top End Health Service, NT Darwin, 0800 Australia

**Keywords:** Bio-feedback, Kegel, Physiotherapist, Stress urinary incontinence, Women’s health

## Abstract

**Introduction and hypothesis:**

The objective was to investigate and compare the efficacy of supervised Kegel exercises with bio-feedback on stress urinary incontinence (SUI) and pelvic floor muscle strength (PFMS) compared with unsupervised Kegel exercises.

**Method:**

Matched-group quasi-experimental study of 29 female participants divided into two groups (supervised and non-supervised) was conducted over 12 weeks. Baseline measurements of PFMS were undertaken by a women’s health physiotherapist and a Kegel exercise regime bespoke designed for each participant. The supervised group visited the physiotherapist monthly for bio-feedback training (BT); the unsupervised group continued at home with their individualised Kegel exercises. Data were collected via a perineometer (Peritron™) and self-reporting responses to questionnaires. All participants received a final PFMS measurement on completion of the study.

**Results:**

Overall Incontinence Severity index (ISI) score was significantly lower in the supervised group post-intervention. Wilcoxon signed-rank tests indicated that supervised Kegel exercises significantly reduced frequency (*p*= 0.002) and severity (*p*= 0.020) of overall ISI. Analysis of PFMS were not significantly different, despite an increase in maximum voluntary contraction or pelvic floor muscle strength (PFMS) (*p*= 0.032) in the supervised group. Of the questionnaires, results of Wilcoxon signed-rank tests indicated that “total bother” was significantly reduced (*p*= 0.005) in the supervised group. The correlation analysis between PFMS and ISI did not reveal any significant results.

**Conclusions:**

The study confirmed that supervised BT is more effective in reducing SUI than unsupervised Kegel exercises, and that this reduction in ISI score did not correlate with the improvement in PFMS.

## Introduction

Stress urinary incontinence (SUI) is the most prevalent form of urinary incontinence and is defined by the International Continence Society (ICS) as a complaint of involuntary loss of urine on effort or physical exertion, excluding sporting activities, or on sneezing or coughing [1]. SUI reportedly affects 25–70% of women worldwide [2]. Women may not disclose this information freely owing to its intimate or embarrassing nature, or to a belief that leakage of urine is a normal part of ageing and not a disease entity [3]; therefore, the accuracy of results is questionable and likely under-reported, with the proportion of women who do not seek professional help reported to be as high as 93% [4]. SUI has a significant impact on women’s mental health, with examples in the literature of depression, anxiety, poor quality of life, low self-esteem, relationship issues related to their sex life [5] and reduced workplace productivity [6].

Almost universally recommended for women with SUI is the conservative management of Kegel exercises, also known as pelvic floor muscle exercises or training. Kegel exercises are repetitive actions of contracting and relaxing the pelvic floor muscles and remain one of the first-line treatments as they are non-invasive, have no known serious side effects and are cost effective [7]. Kegel exercises can be performed from several different positions, such as lying down, standing, or even sitting, providing a variety of convenient choices for the consumer. Further, no equipment is needed to perform Kegel exercises.

Success rates in reducing SUI with the use of Kegel exercises have been cited as ranging from 27% [8] to 75% [9]. The function of the pelvic floor muscles (PFMs) in supporting pelvic structures is pivotal to maintaining continence [10], as leakage results from a weakness or inadequate positioning of the pelvic floor.

Evidence suggests that Kegel exercises are effective; however, this is only true when they are performed correctly. Exploration of effective programmes to strengthen the pelvic floor is vital given that 25–50% of women are unable to correctly activate their pelvic floor [11]. Half of all women attempting Kegel exercises with the aid of a pamphlet are performing this technique incorrectly or reducing the effect with errors of co-contracting the gluteal muscles, the hips and/or the abdominal muscles [8, 12]. Simple verbal or written instructions are not adequate preparation for a pelvic floor exercise programme [12].

Information regarding bio-feedback training (BT) is inconsistent in the literature. Reports range from no statistical difference [13] to improvement in pelvic floor muscle strength (PFMS) over a period of time and “significant” improvements after a 12-week programme [14]; almost 60% of participants in another study required no further therapy owing to the success of treatment with BT as an adjunct to Kegel exercises [15].

The primary goal of this study was to determine the effectiveness on SUI of supervised Kegel exercises using BT versus unsupervised Kegel exercises without BT. A secondary outcome was to establish whether an improvement was observed in PFMS and subsequently, the effect of this on SUI. It was hypothesized that regular support and visual motivation and direction of BT from the physiotherapist might provide greater results.

## Materials and methods

### Study design

**Fig. 1:**
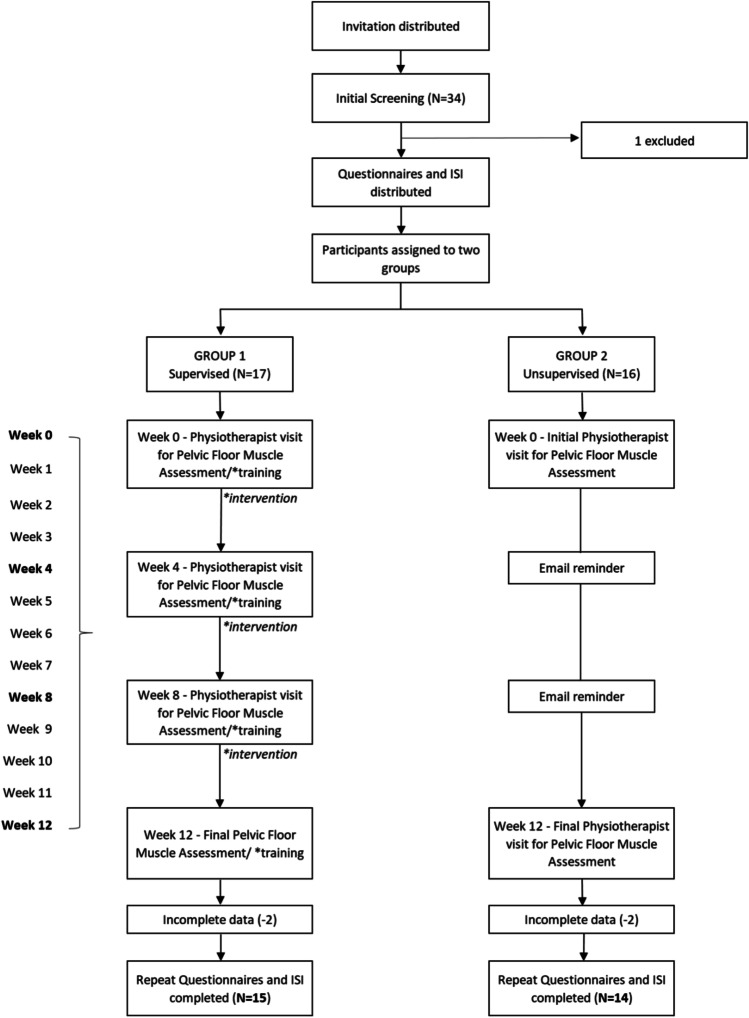
Summary of study protocol. *ISI* Incontinence Severity Index

In this 12-week matched group design, the efficacy of augmented feedback on PFMS and SUI was investigated in women with self-reported SUI; it did not include mixed UI or other (Fig. 1).

### Participants

Participants were recruited from the community via flyers/posters in local shopping and community centres, social media and around a university campus. Participants were initially screened for eligibility on meeting the criteria of being female, aged over 18 years and suffering from SUI (their subjective disclosure was based on a description provided for their reference). They could not be pregnant or breastfeeding for the duration of the study, and they had not performed Kegel exercises. Participants had to be available for the duration of the 12-week programme. The researchers explained the project, benefits and risk to participants and signed informed consent was obtained.

Based on an a priori sample size calculation (G*Power), a sample size of 20 in total was sufficient to detect a significant change in Incontinence Severity Index (ISI) as the main dependent variable when the effect size was set to be moderate, power at 0.80 and alpha level 0.05. Twenty-nine (29) participants completed the study.

This study was approved by the relevant Human Ethics Committee at a local university and all stages of this study was conducted in accordance with the Declaration of Helsinki.

### Pelvic floor assessments

Pelvic floor assessments by a women’s health physiotherapist included an initial 1-h visit where an obstetric/gynaecological history was collected and participants' specific triggers for UI (for example, sneezing, running, coughing etc.) were documented; this information was used to confirm that the participant was suffering from SUI. The procedure was explained to participants, with written and verbal consent obtained for the vaginal (pelvic floor) examination. All participants were then provided with a bespoke Kegel exercise regime.

### Perineometry

The strength of the PFMs was measured using a perineometer (Peritron™; Cardio-Design, Oakleigh, VIC, Australia) and a vaginal sensor. The air-filled silicone rubber sensor was connected to the Peritron™ and inserted vaginally to ascertain a resting baseline PFMS (or pressure) with no squeezing; this was measured in cm/H_2_O and was in place for a minute prior to measurement. Five further measurements were taken; readings were registered by contraction of muscles pushing on the sensor and a maximum measurement taken after 5 s; the sensor was reset to zero between each measurement, with a resting time of 30 s in between to avoid fatigue. The five measurements provided an “average” measurement to be determined. A further 80% maximum voluntary contraction (MVC) pressure and time of hold, up to 20 s, was recorded. During measurements, participants were asked to “squeeze and lift” their pelvic floor, “hold” the contraction and avoid using co-contractions in the abdominal muscles, gluteal muscles or hips. If a participant was unable to activate their pelvic floor, they were excluded from the study. As perineometry is an established technique used by physiotherapists [10], the Peritron™ was chosen for its validity and proven reliability [16]; in this study it guaranteed consistent and reliable quantitative data and ensured intervention fidelity.

### Digital palpation

A pelvic floor examination was conducted with the participant lying supine and her head elevated on two pillows. Using digital palpation, the physiotherapist felt for tone, atrophy and contraction/relaxation of this specific muscle group. Digital palpation was used, as currently there is no tool that provides a comprehensive assessment of the strength and function of the PFMs [17]. This assessment was conducted using a simple and reliable framework developed to evaluate PFMs called the PERFECT scheme. The acronym describes a method of evaluating the Power (pressure), Endurance, Repetitions and Fast contractions) with Every Contraction being Timed of the PFM. This evaluation enabled development of a patient-specific exercise programme based on the severity of muscle weakness. The validity of the PERFECT scheme is supported in studies where treatment using this assessment has produced improvement of SUI in women [18].

### Other assessment

All participants were assessed by a Registered Nurse (RN) as part of the screening process where measurements of heart rate and blood pressure were taken to ensure that these were within normal parameters; additional data on height, weight and body composition analysis were also attained.

### Pelvic floor exercise/training

All participants were provided with a tailored Kegel exercise prescription with a focus on strengthening, relaxation, timing or maintenance. They were advised of the frequency required and a position unique to themselves, to do these exercises; for example, three sets of 10, 5 days per week crook lying (knees bent) on a yoga mat with the aim of holding a contraction for 5 s. Participants were encouraged to find a trigger as a reminder to do exercises such as a phone alarm, when brushing teeth, before bed etc. Supplemental pamphlets available on-line from the International Urogynecological Association (IUGA) such as “Pelvic Floor Exercises: a guide for women” were provided as instructional education. The unsupervised group did not receive any BT during the initial assessment; however, they received a monthly email reminder to perform their Kegel exercises as per their individual programme and return in 12 weeks.

At the initial assessment visit, and subsequent visits, the supervised group had the opportunity of BT by practising their Kegel exercises with the Peritron™ in place receiving professional advice and training based on this visual bio-feedback. All participants in the supervised group attended monthly visits with the physiotherapist, receiving consistent BT to ensure correct pelvic floor activation. Both supervised and unsupervised groups returned in week 12 for a final PFMS assessment.

### Questionnaire tools

Participants in both groups completed multiple questionnaires exploring elements of quality of life in relation to SUI, before and after the intervention period. The first questionnaire provided a baseline calculation of the degree of incontinence measured using the ISI, a validated tool that provides a numerical measure based on the frequency and quantity of urine leakage; the ISI has consistently displayed good criterion validity [19].

Other validated questionnaires utilised to explore the impact of SUI on activities of daily life and quality of life included short versions of the Urogenital Distress Inventory (UDI-6) and the Incontinence Impact Questionnaire (IIQ-7), which evaluate symptom distress and daily life impact; these were included as they have been tested as reliable tools for assessment [20]. The Pelvic Floor Bother Questionnaire (PFBQ) was included for its assessment of pelvic floor disorders and identifying the severity of “bother” of SUI [21].

### Statistical analysis

Data were analysed using IBM Statistical Package for the Social Sciences (SPSS) version 26 (Armonk, NY, USA). The analysis of the data using a Shapiro–Wilk test indicated that many dependent variables in this study were not normally distributed. Hence, the comparison of the differences between groups (supervised vs unsupervised), were performed using Kruskal–Wallis H test. To analyse the difference between pre-intervention and post-intervention, a Wilcoxon signed-rank test was used. The relationship between changes in PFMS and the ISI was investigated using Pearson’s correlation.

## Results

Thirty-four (34) participants met the eligibility criteria. One participant was excluded owing to misunderstanding the criteria (only SUI), 4 participants, 2 from each group, were unable to complete the study; 3 of these were COVID-19 related and the other had unexpected work commitments. The remaining 29 participants, aged 28 to 80, and with a body mass index (BMI) ranging between 17 and 34.3kg/m^2^, and births (parity) ranging from 0 to 5, were divided into supervised (*n*=15) and unsupervised (*n*=14) groups established on their baseline measurements from the total ISI and their age, for as evenly comparative groups as possible. Table 1 presents independent *t* test results to ascertain whether measurements differ between supervised and unsupervised participants; none of these differences reached significance.

**Table 1:** Mean age, body mass index (*BMI*), parity and Incontinence Severity Index (*ISI*) in the supervised and unsupervised groups

	Supervised (*n*=15)	Unsupervised (*n*=14)	*t* value	*p* value
Age	52.50	52.14	−0.16	0.88
BMI	26.08	25.95	0.07	0.94
Parity	2.07	1.79	0.52	0.61
ISI total	4.87	4.50	0.32	0.75

The results indicated that the differences between the supervised and unsupervised groups before intervention were not statistically significant for any variables (*p*>0.05). However, after the intervention period, the ISI score was significantly lower in the supervised group (mean rank=11.97) than in the unsupervised group (mean rank=18.25, *H*=4.193, df=1, *p*=0.041, Cohen’s f=0.420). There were no statistically significant differences in the severity and frequency of SUI between the two groups post-intervention (*p*>0.05). The summary of changes in the two groups and the effect sizes are listed in Table 2.

**Table 2:** Dependent variables, before and after the intervention period in the supervised and unsupervised groups

	Supervised (*n*=15)			Unsupervised (*n*=14)		
	Pre-intervention	Post-intervention	ES	Pre-intervention	Post-intervention	ES
ISI total score	4.87±3.42	2.33±1.95*, **	−0.91	4.50±2.68	3.57±1.95	−0.4
Frequency	2.60±0.83	1.67±0.72**	−1.2	2.57±0.85	2.21±0.97	−0.39
Severity	1.73±0.80	1.27±0.59**	−0.66	1.71±0.61	1.50±0.65	−0.34
PF_rest (cm H_2_O)	29.17±9.59	27.35±9.14	0.19	36.71±13.61	35.44±13.65	0.09
PF_MVC (Avg)	32.33±17.32	43.63±30.20**	−0.46	38.64±27.30	39.02±20.87	−0.02
Time at 80%	10.27±7.35	15.14±6.11**	−0.72	13.71±7.72	13.50±7.35	0.03
UDI-6	51.11±18.04	40.55±12.45**	−0.68	50.90±10.47	43.15±9.18*	−0.79
IIQ-7	17.45±21.19	5.72±9.55**	−0.71	27.21±23.73	14.96±13.17*	−0.64
PFBQ	31.41±15.28	15.99±10.87**	−1.16	31.60±13.67	25.24±11.75	−0.5

*ICI* Incontinence Severity Index, *MVC* maximal voluntary contraction, *PF* pelvic floor, *UDI-6* Urinary Distress Inventory short form, *IIQ-7* Incontinence Impact Questionnaire short form, *PFBQ* Pelvic Floor Bother Questionnaire, *ES* effect size

*Significantly different from the unsupervised group

**Significantly different from pre-intervention

The results of Wilcoxon signed-rank tests indicated that supervised Kegel exercises significantly reduced the frequency (*T*=66, *z*=−3.071, *N*-Ties=11, *p*=0.002, two-tailed); severity (*T*=21, *z*=−2.333, *N*-Ties= 6, *p*= 0.020, two-tailed); and overall ISI score (*T*=66, *z*=−2.965, *N*-Ties= 11, *p*= 0.003, two-tailed). However, changes in the unsupervised group did not reach statistically significant levels.

Additionally, the analysis of pelvic floor functions, including resting pelvic floor pressure, MVC pressure, and 80% of MVCs, showed no statistically significant differences between the two groups after the intervention period. The results of Wilcoxon signed-rank tests indicated that supervised Kegel exercises did not have a statistically significant effect on resting PFM pressure. However, there was a significant increase in MVC PFM in the supervised group (*T*=105, *z*=2.556, *N*-Ties= 15, *p*= 0.011, two-tailed); and 80% of MVCs (*T*=48.5, *z*=2.145, *N*-Ties= 10, *p*= 0.032, two-tailed). Interestingly, these variables did not statistically change in response to the intervention in the unsupervised group.

The analysis of questionnaires further revealed statistically significant difference between the groups after the intervention period. The results of Wilcoxon signed-rank tests indicated that supervised Kegel exercises significantly reduced “total bother” (*T*=109.5, *z*=−2.813, *N*-Ties= 15, *p*= 0.005, two-tailed); IIQ-7 (*T*=79, *z*=−2.345, *N*-Ties= 13, *p*= 0.019, two-tailed); and UDI-6 (*T*=66, *z*=−2.946, *N*-Ties= 11, *p*= 0.003, two-tailed). In the unsupervised group, the “total bother” score did not significantly change in response to the intervention; however, there was a significant reduction in the IIQ-7 (*T*=45, *z*=−2.670, *N*-Ties= 9, *p*= 0.008, two-tailed) and in the UDI-6 (*T*=65, *z*=−2.852, *N*-Ties= 11, *p*= 0.004, two-tailed).

The analysis of the relationship between PFMS and the ISI did not reveal any significant correlation between changes in PFMS and ISI. Similarly, the relationships between PFMS and ISI did not reach a level of significance before or after the intervention period.

Adverse effects were negated by having multiple sensors with a strict one-participant/one-sensor regime for the entire study to eliminate the risk of cross-contamination, by stringent infection controls and by an opt-out for participants if they were distressed at any point.

## Discussion

This study investigated the efficacy of supervised and unsupervised Kegel exercises on UI and PFMS amongst women with SUI. We hypothesised that supervised Kegel exercises, compared with the unsupervised approach, result in a greater reduction of SUI and improvement of PFMS. The results of this study supported the hypothesis and confirmed that despite an improvement of SUI and PFMS in both groups, supervised Kegel exercises with BT is more effective in reducing UI amongst women who suffer from chronic SUI.

The improvement in the supervised group can be attributed to regularly visiting a women’s health physiotherapist and receiving BT. This suggestion is supported in findings in the literature that provision of regular/consistent supervision and professional guidance yields a better result [22, 23]. In a similar study, it was found that PFMS improved with or without BT and that it may not be necessary; however, it could be offered as an adjunct therapy [24]. Further to this, in a study in which all participants were offered BT as an adjunct to their Kegel exercises, almost 60% required no further therapy after 3 months [15]. In our study, participants in the supervised group visited a women’s health physiotherapist every 4 weeks over a 3 month period for training and BT, which proved sufficient for a positive outcome. It is plausible that receiving feedback was more motivating for the supervised group and encouraged compliance with their programme of Kegel exercises.

There are mixed results in the literature regarding bio-feedback; however, there is a propensity to include this tool when a physiotherapist assesses and trains individuals to correctly activate pelvic floor muscles, as was done in the supervised group in this study. Bio-feedback has a place in supporting the teaching of how to correctly contract pelvic floor muscles, in conjunction with verbal coaching and supervision by an appropriate therapist [13]. This study agreed with many, that PFMS can be improved with Kegel exercises. However, the degree of improvement in the supervised group with BT, was considerable in comparison, as was the improvement in severity and frequency of SUI. This led to the conclusion that supervision with BT is imperative to facilitate success in these programmes and would be a recommended course of action for women suffering from SUI.

Despite the effectiveness of bio-feedback in reducing SUI in many studies, the practice can be costly, time consuming and may not be available to remote locations or areas with limited access to health professionals. The results of our study showed that women with SUI can follow personalised training instructions after the first visit to a physiotherapist, or pelvic floor specialist, and can benefit from self-managed Kegel exercises. For women unable to regularly access a physiotherapist, this treatment modality may be initially sufficient.

The results of this study further indicated an improvement in average measurement of pelvic floor resting pressure after 12 weeks in both groups even though this was not statistically significant. Although the resting pelvic floor pressure did not show a statistically significant improvement, the MVC improved significantly in the supervised group. The MVC, measured during a pelvic floor contraction, results from activation of multiple muscle fibres; the more muscle fibres activated, the stronger the contraction. The ability to contract the pelvic floor muscles correctly and observing results can influence adherence, as shown by the significant positive effects in our supervised group.

Even though the degree of MVC improvement between supervised and unsupervised groups was not significant, the supervised group did show more improvement. The lesser degree of improvement in the unsupervised group could be due to an incorrect technique being performed or an inability to isolate or contract pelvic floor muscles. Bo [25] found that more than 30% of women do not contract their pelvic floor muscles correctly when performing Kegel exercises, using other accessory muscles instead, and further, up to 50% of women who attempt to learn the exercises with the aid of a pamphlet, get the technique wrong [26]. Lack of knowledge about how to perform Kegel exercises was found to be a barrier and participants confirmed that seeking information from the internet or pamphlets was not sufficient, although these are often provided as active treatment [27].

Significant results were obtained from our 12-week study; however, considerable improvement in SUI was seen in another study after only 4 weeks of BT with a physiotherapist [28]. It is suggested that societal education might be required regarding the beneficial role of a women’s health physiotherapist, as it seems that they are often overlooked as a source of education and treatment for UI. This would be particularly valuable for remote/non-urban populations, where physiotherapists/specialists visit irregularly, or for women who may not have the financial freedom of ongoing regular physiotherapist visits.

This study shares the opinion that Kegel exercises are vital for the improvement of PFMS and further recommend the benefit of BT from a women’s health physiotherapist, if only initially for a short period, for brief training and re-education of efficient pelvic floor muscle contraction. Although SUI in the supervised group improved more considerably, the education and individualised Kegel exercise programme designed for each of the participants at the beginning of the study, awareness/education of pelvic floor muscles and increased strength in contractions may have contributed to the success.

It is proposed that there is a distinct lack of relationship between the PFMS and ISI. This study has demonstrated that SUI has improved in most cases, although the resting pelvic floor muscle pressure has not shown a significant improvement. However, the relationship between the strength in muscle contraction and reduced SUI is significant—more so in the supervised group—leading to the conclusion that perhaps the ability to perform Kegel exercises correctly (supervised) is the beneficial factor. This study strongly supports BT with a physiotherapist and a bespoke Kegel exercise programme for treatment of SUI. It is acknowledged, however, that even one visit with BT and provision of an individualised Kegel exercise programme would be valuable, and, with compliance, could prove transformative to women who are unable to frequently access a physiotherapist.

The use of questionnaires to determine quality of life should be considered as an adjunct for the health professional when determining the success of an intervention. In our study, self-reporting in questionnaires pre– and post-intervention yielded significant increases in subjective improvement in the bother and incidence of SUI. The supervised group indicated significant improvements to their SUI in three surveys, whereas the unsupervised group indicated improvement in two. Increasing severity of SUI appears to correlate with a decrease in quality of life and this is reflected throughout the literature. It could be speculated that the self-reported improvement in SUI, and improvement in quality of life, may have been achieved by actively participating in the study; however, the data conclusively indicate a physical improvement in SUI.

Self-reporting questionnaires such as the PFBQ, UDI-6 and IIQ-7 have proven valuable tools for determining improvements in SUI in women. The IIQ-7 and the UDI-6 specifically, are effective when used together [29], to determine the impact of SUI on a person’s life. Participants in this study recorded improvements in SUI reflecting improved quality of life.

Results from the PFBQ indicated an improvement in the supervised group only, whereas data from the UDI-6 with a focus on the severity of UI, and the IIQ-7, concentrating on the impact of UI on activities and emotions in general, were reported to have demonstrated a significant change in both groups. This study affirms that self-reporting questionnaires are a valuable adjunct to supporting the physical data from pelvic floor muscle assessments, with improvements in quality of life indicated in all questionnaires.

The authors acknowledge that this study had some limitations. First, the sample size was relatively small and based on a convenience sample in an urban environment. Second, despite monthly email contact with the unsupervised group, compliance with the Kegel exercises was not determined exactly. Last, a greater MVC in the supervised group may be attributed to a learning effect and not necessarily to an increase in PFMS.

## Conclusion

This study confirmed that Kegel exercises are important and effective when performed correctly, and the inclusion of supervised BT was found to be a significant indicator of improving incontinence in women suffering from SUI. The addition of a women’s health physiotherapist to train and adjust the technique contributed to the participants being able to perform the Kegel exercises correctly. It is considered that the reduction in SUI is attributed to the significant improvement in the ability to promptly and effectively activate pelvic floor muscles and is not necessarily related to the PFMS.

The following recommendations are made. Based on the positive results of BT from this study, it is recommended that at the very least, brief BT training for the initial modification/re-education of correct pelvic floor muscle contraction, in addition to a bespoke Kegel exercise regime, would be the ideal treatment for SUI. Results from this study are positive and encouraging; however, further research is needed into other modalities that may also improve the PFMS and as a result, reduce SUI, as BT, a physiotherapist or continence nurse may not always be accessible to women, depending on location. Additionally, the cost of treatment, how many treatments are required and how often BT is needed warrant further research.

## References

[1] D'Ancona C, Haylen B, Oelke M (2019). The International Continence Society (ICS) report on the terminology for adult male lower urinary tract and pelvic floor symptoms and dysfunction. Neurourol Urodyn.

[2] Milsom I, Gyhagen M (2019). The prevalence of urinary incontinence. Climacteric.

[3] Yip SK, Cardozo L (2007). Psychological morbidity and female urinary incontinence. Best Pract Res Clin Obstet Gynecol.

[4] Minassian VA, Drutz HP, Al-Badr A (2003). Urinary incontinence as a worldwide problem. Int J Gynecol Obstet.

[5] Caruso S, Brescia R, Matarazzo MG, Giunta G, Rapisarda AMC, Cianci A (2017). Effects of urinary incontinence subtypes on women's sexual function and quality of life. Urology.

[6] Fultz N, Girts T, Kinchen K, Nygaard I, Pohl G, Sternfeld B (2005). Prevalence, management and impact of urinary incontinence in the workplace. Occup Med (Lond).

[7] Price N, Dawood R, Jackson SR (2010). Pelvic floor exercise for urinary incontinence: a systematic literature review. Maturitas.

[8] Newman DK, Wein AJ (2013). Office-based behavioral therapy for management of incontinence and other pelvic disorders. Urol Clin North Am.

[9] Bo K, Morkved S, Frawley H, Sherburn M (2009). Evidence for benefit of transversus abdominis training alone or in combination with pelvic floor muscle training to treat female urinary incontinence: a systematic review. Neurourol Urodyn.

[10] Quartly E, Hallam T, Kilbreath S, Refshauge K (2010). Strength and endurance of the pelvic floor muscles in continent women: an observational study. Physiotherapy.

[11] Barton A, Serrao C, Thompson J, Briffa K (2015). Transabdominal ultrasound to assess pelvic floor muscle performance during abdominal curl in exercising women. Int Urogynecol J.

[12] Bump RC, Hurt WG, Fantl JA, Wyman JF (1991). Assessment of Kegel pelvic muscle exercise performance after brief verbal instruction. Am J Obstet Gynecol.

[13] Morkved S, Bo K, Fjortoft T (2002). Effect of adding biofeedback to pelvic floor muscle training to treat urodynamic stress incontinence. Obstet Gynecol.

[14] Lee HN, Lee SY, Lee YS, Han JY, Choo MS, Lee KS (2013). Pelvic floor muscle training using an extracorporeal biofeedback device for female stress urinary incontinence. Int Urogynecol J.

[15] Yoo EH, Kim YM, Kim D (2011). Factors predicting the response to biofeedback-assisted pelvic floor muscle training for urinary incontinence. Int J Gynaecol Obstet.

[16] Frawley HC, Galea MP, Phillips BA, Sherburn M, Bo K (2006). Reliability of pelvic floor muscle strength assessment using different test positions and tools. Neurourol Urodyn.

[17] Bo K, Sherburn M (2005). Evaluation of female pelvic-floor muscle function and strength. Phys Ther.

[18] Oliveira Camargo F, Rodrigues A, Arruda M, Ferreira Sartori R, Girão M, Castro M (2009). Pelvic floor muscle training in female stress urinary incontinence: comparison between group training and individual treatment using PERFECT assessment scheme. Int Urogynecol J.

[19] Sandvik H, Espuna M, Hunskaar S (2006). Validity of the incontinence severity index: comparison with pad-weighing tests. Int Urogynecol J Pelvic Floor Dysfunct.

[20] Utomo E, Korfage IJ, Wildhagen MF, Steensma AB, Bangma CH, Blok BF (2015). Validation of the Urogenital Distress Inventory (UDI-6) and Incontinence Impact Questionnaire (IIQ-7) in a Dutch population. Neurourol Urodyn.

[21] Peterson TV, Karp DR, Aguilar VC, Davila GW (2010). Validation of a global pelvic floor symptom bother questionnaire. Int Urogynecol J.

[22] Hay-Smith EJ, Herderschee R, Dumoulin C, Herbison GP (2011). Comparisons of approaches to pelvic floor muscle training for urinary incontinence in women. Cochrane Database Syst Rev.

[23] Konstantinidou E, Apostolidis A, Kondelidis N, Tsimtsiou Z, Hatzichristou D, Ioannides E (2007). Short-term efficacy of group pelvic floor training under intensive supervision versus unsupervised home training for female stress urinary incontinence: a randomized pilot study. Neurourol Urodyn.

[24] Hirakawa T, Suzuki S, Kato K, Gotoh M, Yoshikawa Y (2013). Randomized controlled trial of pelvic floor muscle training with or without biofeedback for urinary incontinence. Int Urogynecol J.

[25] Bo K (2003). Pelvic floor muscle strength and response to pelvic floor muscle training for stress urinary incontinence. Neurourol Urodyn.

[26] Continence Foundation of Australia. Pelvic floor muscles/common myths 2018. Available from: https://www.continence.org.au/pages/common-myths-about-your-pelvic-floor.html. Accessed 23 Feb 2020

[27] Sjostrom M, Umefjord SH, Carlbring P, Andersson G, Samuelsson E (2015). Internet-based treatment of stress urinary incontinence: 1- and 2-year results of a randomized controlled trial with a focus on pelvic floor muscle training. BJU Int.

[28] Ronak S, Priyanshu R, Neeta J (2014). The efficacy of physiotherapy management in women with stress urinary incontinence—pilot study. Indian J Physiother Occup Ther.

[29] Nie XF, Ouyang YQ, Wang L, Redding SR (2017). A meta-analysis of pelvic floor muscle training for the treatment of urinary incontinence. Int J Gynaecol Obstet.

